# The Effect of Fixed Orthodontic Appliances and Fluoride Mouthwash on the Oral Microbiome of Adolescents – A Randomized Controlled Clinical Trial

**DOI:** 10.1371/journal.pone.0137318

**Published:** 2015-09-02

**Authors:** Jessica E. Koopman, Nicoline C. W. van der Kaaij, Mark J. Buijs, Yassaman Elyassi, Monique H. van der Veen, Wim Crielaard, Jacob M. ten Cate, Egija Zaura

**Affiliations:** 1 Department of Preventive Dentistry, Academic Centre for Dentistry Amsterdam, Amsterdam, The Netherlands; 2 Department of Orthodontics, Academic Centre for Dentistry Amsterdam, Amsterdam, The Netherlands; National University of Singapore, SINGAPORE

## Abstract

While the aesthetic effect of orthodontic treatment is clear, the knowledge on how it influences the oral microbiota and the consequential effects on oral health are limited. In this randomized controlled clinical trial we investigated the changes introduced in the oral ecosystem, during and after orthodontic treatment with fixed appliances in combination with or without a fluoride mouthwash, of 10–16.8 year old individuals (N = 91). We followed several clinical parameters in time, in combination with microbiome changes using next-generation sequencing of the bacterial 16S rRNA gene. During the course of our study, the oral microbial community displayed remarkable resilience towards the disturbances it was presented with. The effects of the fluoride mouthwash on the microbial composition were trivial. More pronounced microbial changes were related to gingival health status, orthodontic treatment and time. Periodontal pathogens (e.g. *Selenomonas* and *Porphyromonas*) were highest in abundance during the orthodontic treatment, while the health associated *Streptococcus*, *Rothia* and *Haemophilus* gained abundance towards the end and after the orthodontic treatment. Only minor compositional changes remained in the oral microbiome after the end of treatment. We conclude that, provided proper oral hygiene is maintained, changes in the oral microbiome composition resulting from orthodontic treatment are minimal and do not negatively affect oral health.

## Introduction

The aesthetic effects of orthodontic treatment are often readily visible; in contrast to the effect orthodontic treatment might have on the non-visible part of the oral cavity—the microbiome.

The possible changes in the oral microbiome during orthodontic treatment are likely to be related to, the more easy observable, clinical parameters. For instance, the impaired gingival health status [1, 2] and increased plaque formation [3, 4] that are associated with the placement of fixed orthodontic appliances. Besides, the latter could lead to the formation of white spot lesions, creating an undesirable aesthetic effect and possibly resulting in a cavity in need of restauration [5, 6].

So far, studies aimed to investigate the changes in bacterial taxa during orthodontic treatment, used culturing or targeted molecular approaches, allowing for a limited number of opportunistic pathogenic species to be observed [7–10]. This implies that the response of the entire microbiome to orthodontic treatment is unclear, as are the possible long-term changes in bacterial composition.

A full understanding of the effects of fixed orthodontic appliances on the oral microbiome and the consequences on clinical parameters, should allow for the preservation of a healthy oral cavity during and after orthodontic treatment, justifying orthodontic treatment.

Our aim was to investigate the changes introduced in the oral ecosystem during and after orthodontic treatment in combination with a fluoride mouthwash. To our knowledge, this is the first study to investigate the dynamics of the oral microbiome of adolescents during orthodontic treatment, and the use of a fluoride mouthwash using an open-ended molecular approach.

## Materials and Methods

### Sampling and treatment

A randomized placebo-controlled parallel clinical trial was performed as described by van der Kaaij *et al*. [11]. The study was approved by the Medical Ethical Committee of the VU Medical Centre of the VU University of Amsterdam (VU-METc 2009/026 and Dutch trial register: NTR1817 [12]). The randomization allocation list was made in Microsoft Office Excel 2003 (Microsoft, Redmond, WA, USA) using the random number generation function in the analysis toolpack for one variable with a discrete distribution, allocating 50% of the 120 subjects to the test and 50% to the control group. The study was powered on the basis of the primary outcome; the data presented here were secondary outcomes.

All subjects participating in this study were scheduled to receive full fixed orthodontic appliances. Subjects could only be scheduled to receive full fixed orthodontic appliances if they maintained a proper oral hygiene and had no severe gingivitis. The guidelines at the Orthodontic Department at ACTA state that orthodontic appliances will not be placed when the bleeding by probing score is above 2 (1: 0–5% of the sites are bleeding, 2: 6–10% of the sites are bleeding, 3: 11–20% of the sites are bleeding, 4: 21–35% of the sites are bleeding, 5: > 35% of the sites are bleeding), except if immediate orthodontic treatment is indicated, for example, in case of traumatic occlusion.

The inclusion criteria for the study were: 10–18 yrs of age, good general health, no use of medication and no demineralizations in need of restauration present at a buccal surface, in addition to providing their written informed consent. A total of 120 subjects set to receive fixed orthodontic appliances in both jaws were to participate in the study. Roth Ovation Brackets (Dentsply, GAC International, Bohemia, NY, USA) were used and all were bonded following the same procedure and methods, using Transbond XT primer and adhesive (3M unitek, Monrovia, USA).

In this triple-blind study, the subjects received a randomly assigned mouthwash containing 100 ppm amine-fluoride (AmF) and 150 ppm sodium-fluoride (SnF_2_) (Elmex caries protection, Colgate-Palmolive Europe, Therwil, Switzerland) or a placebo, also provided by Colgate-Palmolive Europe. The mouthwash was used from the time of bonding until debonding. The subjects were instructed not to use fluoride containing products, other than toothpaste, during the course of the study. Their dentist was informed about the study and was asked not to apply extra fluoride during the study period. Furthermore, the subjects received oral hygiene instructions after placement of the fixed appliances and were advised to use interproximal brushes to clean the areas of the tooth adjacent to the bracket underneath the orthodontic wire.

The subjects were instructed not to clean their teeth 24 h before supragingival plaque samples for microbiome analysis were taken. These samples were obtained at six time-points during this study: T0 (approximately one week before placement of the fixed orthodontic appliances), T1 (six weeks after placement), T2 (twelve weeks after placement), TD (debonding, average of 25 months after placement), TD1 (six weeks after debonding) and TD2 (twelve weeks after debonding). Supragingival plaque was collected from the buccal surface of the upper left premolars using a sterile plastic spatula. In presence of the brackets (visits T1, T2 and TD), which were placed on the middle of the tooth, the plaque was collected between the gingiva and the bracket. Gingival swelling often occurs within one or two months after placement of orthodontic appliances [1, 13, 14]. Hence, in cases where the gingival margin reached the bracket, the plaque was collected mesially and/or distally from the bracket. The plaque samples were spun down for 30 s at 16.100 x g and stored at -80°C.

The number of white spot lesions of the subjects was recorded at visits T0, TD, TD1 and TD2, and is described in more detail by van der Kaaij *et al*. [11]. Additionally, a bleeding by probing score was recorded at each visit for each patient. The percentage-based bleeding score was determined by probing each (bonded or to be bonded) tooth mesiobuccally and distobuccally with a periodontal probe [11]. For statistical analysis, the bleeding score was dichotomized into a healthy (score 1) and a gingivitis (score 2–5) group.

### DNA isolation and sequencing

DNA was isolated from the supragingival plaque samples as described by Zaura *et al*. [15]. The V5-V7 regions of the 16S rDNA were used to prepare barcoded amplicon libraries for each sample [16]. The equimolar pooled samples were sequenced at the Academic Medical Center (Amsterdam, the Netherlands) and Macrogen Inc. (Seoul, Republic of Korea) using the 454 FLX Titanium chemistry (Roche, Basel, Switzerland). The reads are available at NCBI’s Sequence Read Archive under SRP055565.

### Sequencing data analysis

Quantitative Insights Into Microbial Ecology (QIIME) v1.5.0 was used to analyze the sequence data [17]. The downstream analyses and clustering into OTUs was done according to Koopman *et al*. [18], with the exception that 1 ambiguous base (N = 1) was allowed. The OTUs were manually aligned against NCBI’s nucleotide (nr/nt) collection using Megablast [19, 20] to obtain species level identification (S1 Table).

### Statistical analysis

The Shannon diversity index and Bray-Curtis similarity index were calculated using PAST v3.0 [21]. This program was also used to construct non-metric multidimensional scaling (nmMDS) plots based on the Bray-Curtis coefficient to visualize similarity between the samples. Stress < 0.2 (Kruskal’s stress formula 1) was used as a threshold [22].

The statistical significance of individual OTUs in relation to clinical parameters was determined using QIIME’s paired t-test and correlation. The OTUs that were significant after FDR correction for multiple comparisons were analyzed further using IBM SPSS Statistics v21 (IBM Corp, Armonk, NY, USA). The Mann-Whitney test was used to determine if there was a statistically significant difference between the mouthwash groups, or gingival health status per visit for the phyla, genera and OTUs. The Wilcoxon Signed Ranks test was used to examine if there was a statistically significant difference between the visits at phylum, genus and OTU level and for the Shannon diversity index.

## Results

### Study population

A total number of 120 subjects participated in the study. Contribution of 22 subjects to this study was discontinued because they declined further participation, moved or failed to show up. For 7 of the subjects, no supragingival plaque samples could be obtained because they brushed their teeth prior to sampling or the quality of the reads after sequencing was poor. From the 91 remaining subjects, one or more supragingival plaque samples were obtained. The number of microbiological samples obtained per visit was: T0; n = 76, T1; n = 73, T2; n = 68, TD; n = 44, TD1; n = 43 and TD2; n = 45. The number of subjects per mouthwash group per visit and the gender ratio per visit are described in S2 Table. At the time of bonding, the average age of the subjects was 13.3 years old (SD 1.4, range 10–16.8). There was no significant difference in gingival bleeding between the group receiving the fluoride mouthwash and the group receiving the placebo at the baseline visit [11].

### Sequencing output

Of the processed sequencing reads, 78% passed quality control and 75% (2607737 reads) remained after the removal of chimeric reads. For 31 of the samples the number of reads was too low (8–769 reads per sample, average 227 reads); these were excluded from further analyses. The remaining 349 samples had an average of 7164 reads per sample (SD 5131, range 835–28432). The reads clustered into 461 OTUs. The subsampling threshold was set at 800 reads and the remaining subset, containing an average of 49 OTUs per sample (SD 14, range 11–94), was used for further analysis.

The reads were classified into 15 phyla and, when averaged over all time-points, dominated by Firmicutes (27%), Actinobacteria (22%), Proteobacteria (22%), Bacteroidetes (16%), Fusobacteria (11%) and Candidate division TM7 (1%). At a lower taxonomic level, the reads were classified into 149 genera, dominated by *Streptococcus* (12%), *Neisseria* (11%), *Corynebacterium* (9%), *Veillonella* (7%), *Leptotrichia* (7%) and *Actinomyces* (6%).

### Mouthwash effect

**Fig 1:**
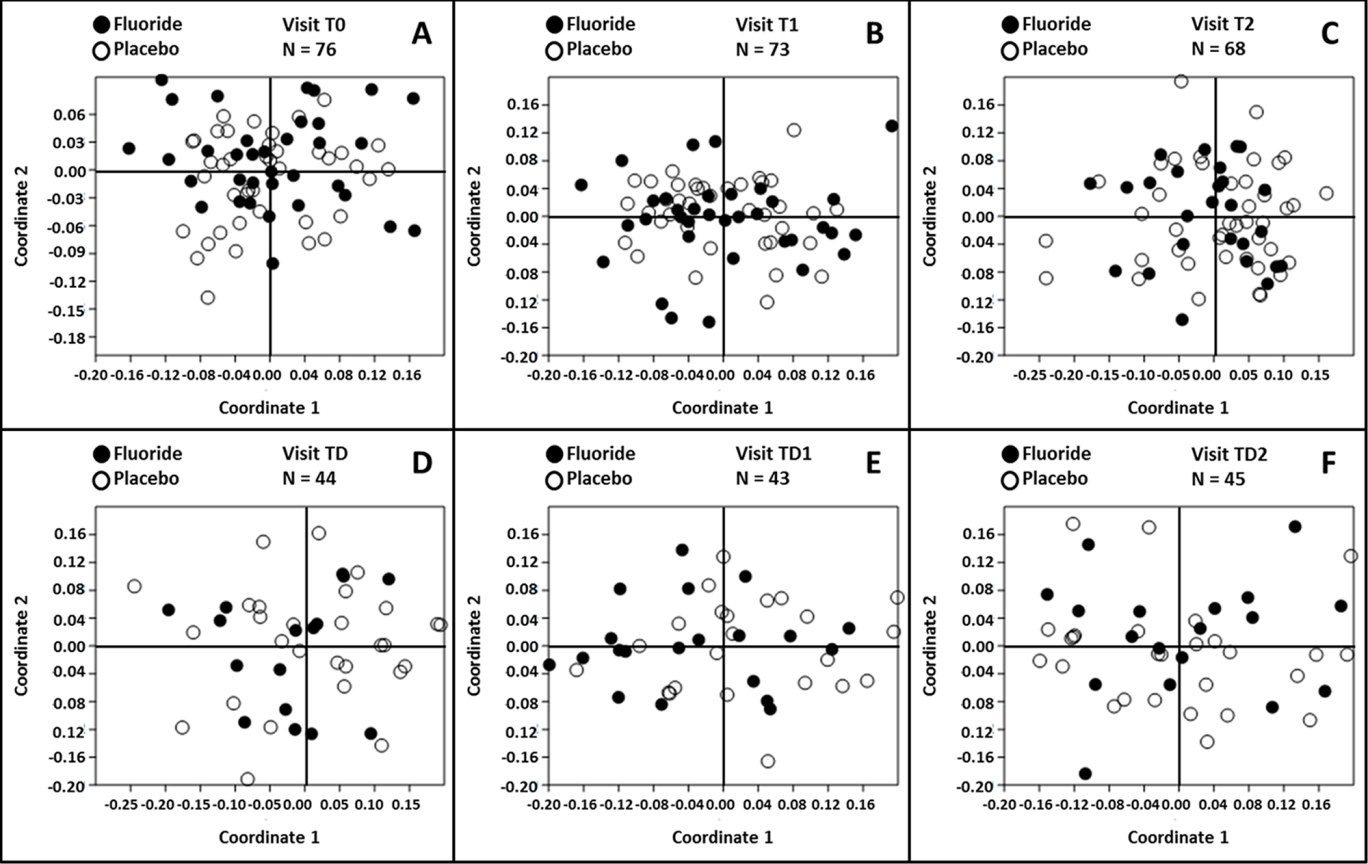
Non-metric multidimensional scaling plots based on the three-dimensional Bray-Curtis similarity index by rinse. The plots are constructed per visit. Subjects receiving rinse A are symbolized by ●, the subject receiving rinse B are indicated with ○. Mouthwashes were administered between visits T0 and TD. The stress for each individual plot is (A) 0.1543, (B) 0.1465, (C) 0.1256, (D) 0.1402, (E) 0.1495 and (F) 0.1531.

Non-metric multidimensional scaling plots were made by mouthwash group per visit. These plots did not show any separation of the microbial profiles based on mouthwash (Fig 1). There were no statistically significant differences in Shannon diversity index at any of the visits. To assess the stability of the microbiome composition in time, the Bray-Curtis similarity index between visit T0 and the subsequent visits was calculated per individual and tested for each mouthwash group. The difference in similarity did not reach statistical significance at any of the time-points.

There was no significant difference in relative abundance of any bacterial phylum between the two mouthwash groups at any visit.

At genus level, within the placebo group, *Fusobacterium* decreased significantly in abundance from visit T0 to T1 (P = 0.049) and from T1 to T2 (P = 0.002). Between visits T2 and TD, the level of abundance became significantly higher again (P = 0.038) (S1 Fig). In the fluoride mouthwash group, there was no significant difference in abundance of *Fusobacterium* between any of the visits (S1 Fig).

At the OTU level, the abundance of OTU381 (*Kingella*) was higher (P = 0.028) in the placebo group compared to the fluoride group at visit T1 (S2 Fig).

### Gingival health

**Fig 2:**
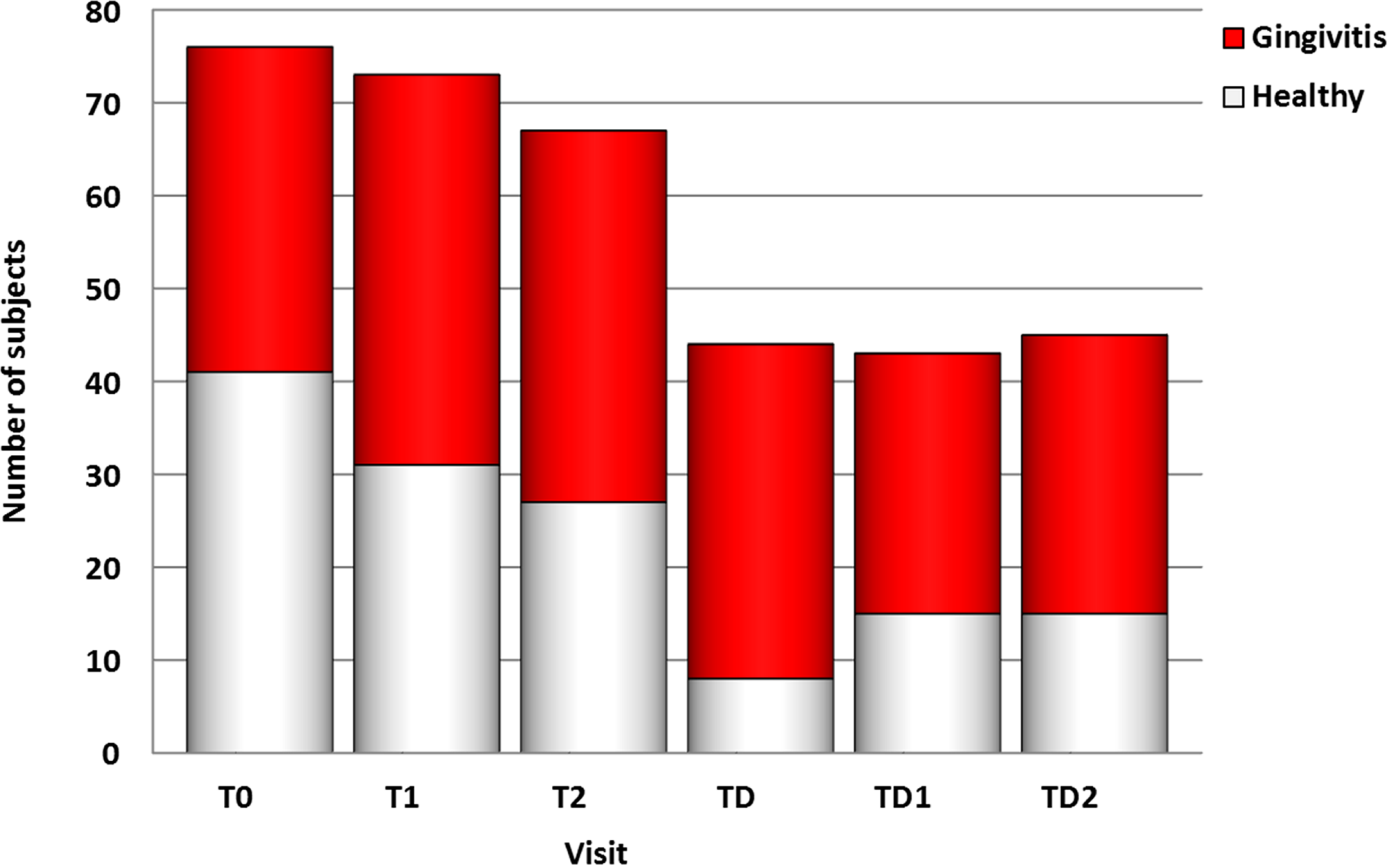
Count of subjects with healthy gingiva and gingivitis per visit.

**Fig 3:**
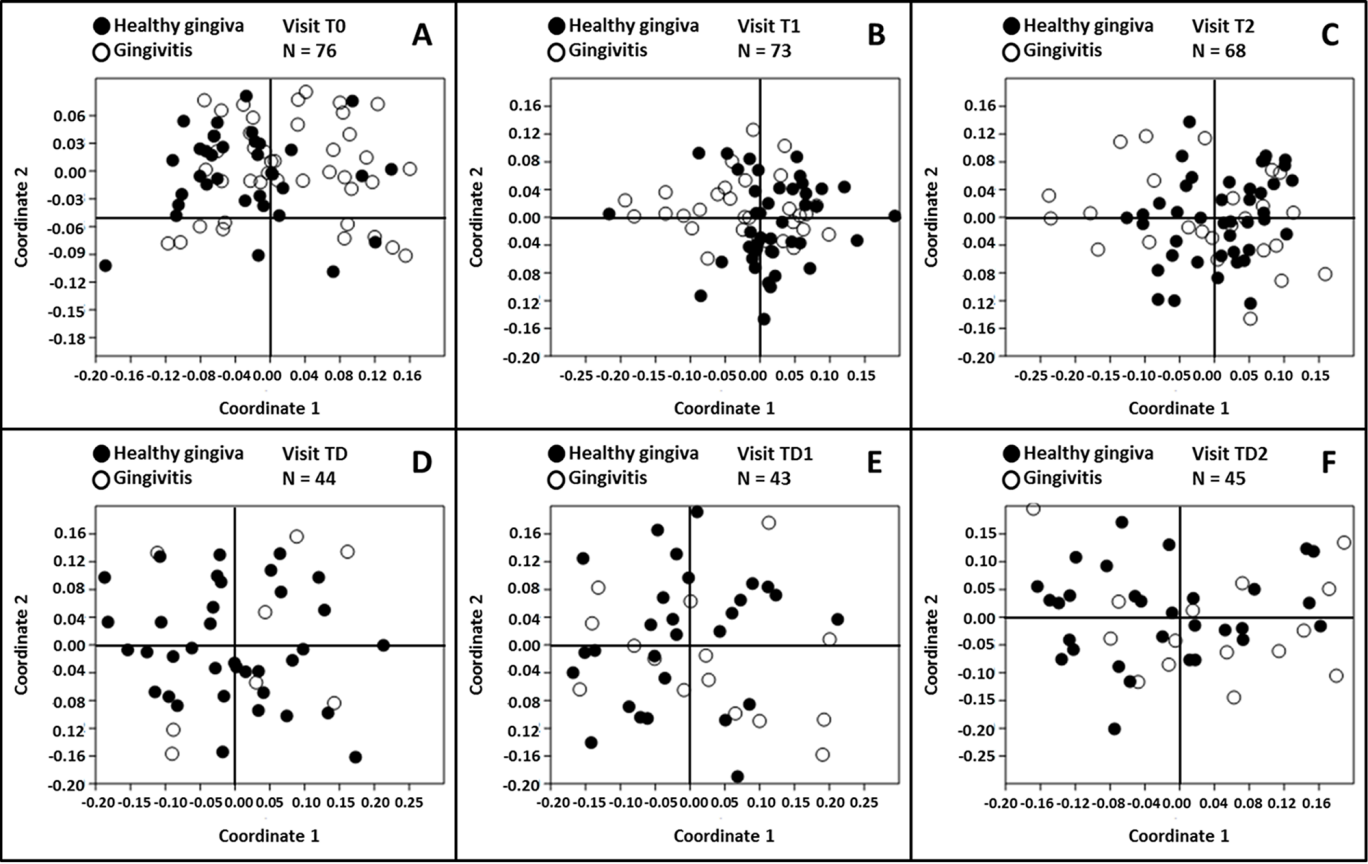
Non-metric multidimensional scaling plots based on the three-dimensional Bray-Curtis similarity index by gingival health status. The plots are constructed per visit. The subjects with healthy gingiva are indicated with ○, the subjects with gingivitis are indicated with ●. *Stress* for the individual plots is (A) 0.1539, (B) 0.1474, (C) 0.1265, (D) 0.1401, (E) 0.1495 and (F) 0.1531.

The gingival health status of the subjects was determined by probing. To assess the relation between gingival health and the supragingival plaque microbiome, we dichotomized the group into subjects with healthy gingiva and with gingivitis. The highest prevalence of gingivitis was recorded at visit TD (Fig 2). Non-metric multidimensional scaling plots based on the OTU profiles of each subject per time-point showed that gingivitis-microbiome profiles were less scattered, especially at visits T0, T1 and T2, in space compared to the healthy-gingiva microbiome profiles (Fig 3).

At the phylum level, the proportion of Bacteroidetes was higher in the individuals with gingivitis compared to those with healthy gingiva at visits T0 (P = 0.012) and T1 (P = 0.035) (S3A Fig). The abundance of Candidate division TM7 was significantly elevated in individuals with gingivitis at visits T0 (P = 0.001), T1 (P = 0.029), T2 (P = 0.032) and TD2 (P = 0.037) (S3B Fig). The proportion of the phylum Fusobacteria was higher in the subjects with gingivitis at visits T1 (P = 0.031) and TD2 (P = 0.024) (S3C Fig).

At genus level, the relative abundance of the genus *Selenomonas* was significantly higher in the gingivitis group compared to the healthy group at visits T0 (P = 0.022), T1 (P = 0.041) and TD2 (P = 0.012) (S4A Fig). The same applied to *Porphyromonas* at visits T0, T1 and T2 (P = 0.036, P = 0.010 and P = 0.033, respectively) (S4B Fig) and *Johnsonella* at visits T0 (P = 0.0040), T1 (P = 0.013) and TD2 (P = 0.042) (S4C Fig). In contrast, the genus *Derxia* was significantly higher in the healthy group at visits T0 and T1 (P = 0.046 and P = 0.028, respectively) (S4D Fig). The same was observed for the genera *Haemophilus* at visit T0 (P = 0.021) and visit TD2 (P = 0.024) (S4E Fig) and *Rothia* at visit T0 (P = 0.004) (S4F Fig).

In agreement with the genus *Rothia*, OTU65 (*Rothia*) was significantly more abundant in the healthy subjects compared to those with gingivitis at visit T0 (P = 0.011) (S5A Fig). The difference in abundance in OTU351 (*Streptococcus*) between the two groups was significant at visit T1 (P = 0.023) where the OTU was higher in number in the healthy group (S5B Fig). On the other hand, OTU424 (*Johnsonella*) was more abundant in the gingivitis group compared to the healthy group at visits T0 (P = 0.032), T1 (P = 0.039) and TD (P = 0.044) (S5C Fig). The OTUs 55, 171 and 355, all three classified as Candidate division TM7, were higher in the gingivitis group at visit T0 (P = 0.005, 0.006 and 0.005, respectively). OTU355 was also higher at T1 (P = 0.011), while OTU55 was higher at visit T2 (P = 0.011) in the gingivitis group (S5D–S5F Fig). The OTU302 (*Selenomonas*) was significantly higher in the gingivitis group compared to the healthy group at T0 (P = 0.038), T1 (P = 0.045) and TD2 (P = 0.010) (S5G Fig) as was OTU398 (*Fusobacterium*) at TD2 (P = 0.012) (S5H Fig).

### Time

**Fig 4:**
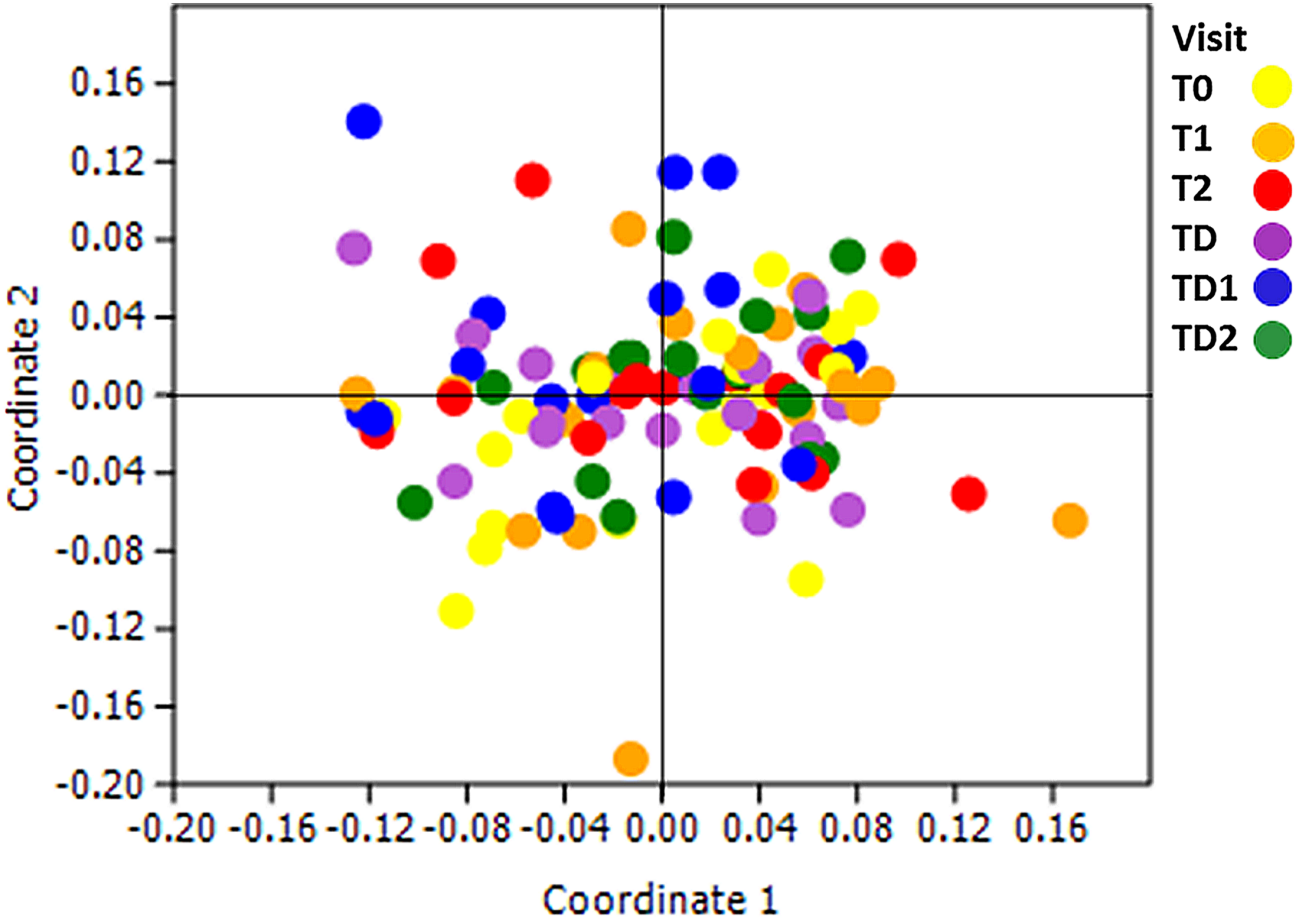
Non-metric multidimensional scaling plot based on the three-dimensional Bray-Curtis similarity index by time. The plot consists of samples of all subjects (N = 19) who were present at all six time points. The *stress* for this plot is 0.1836.

**Fig 5:**
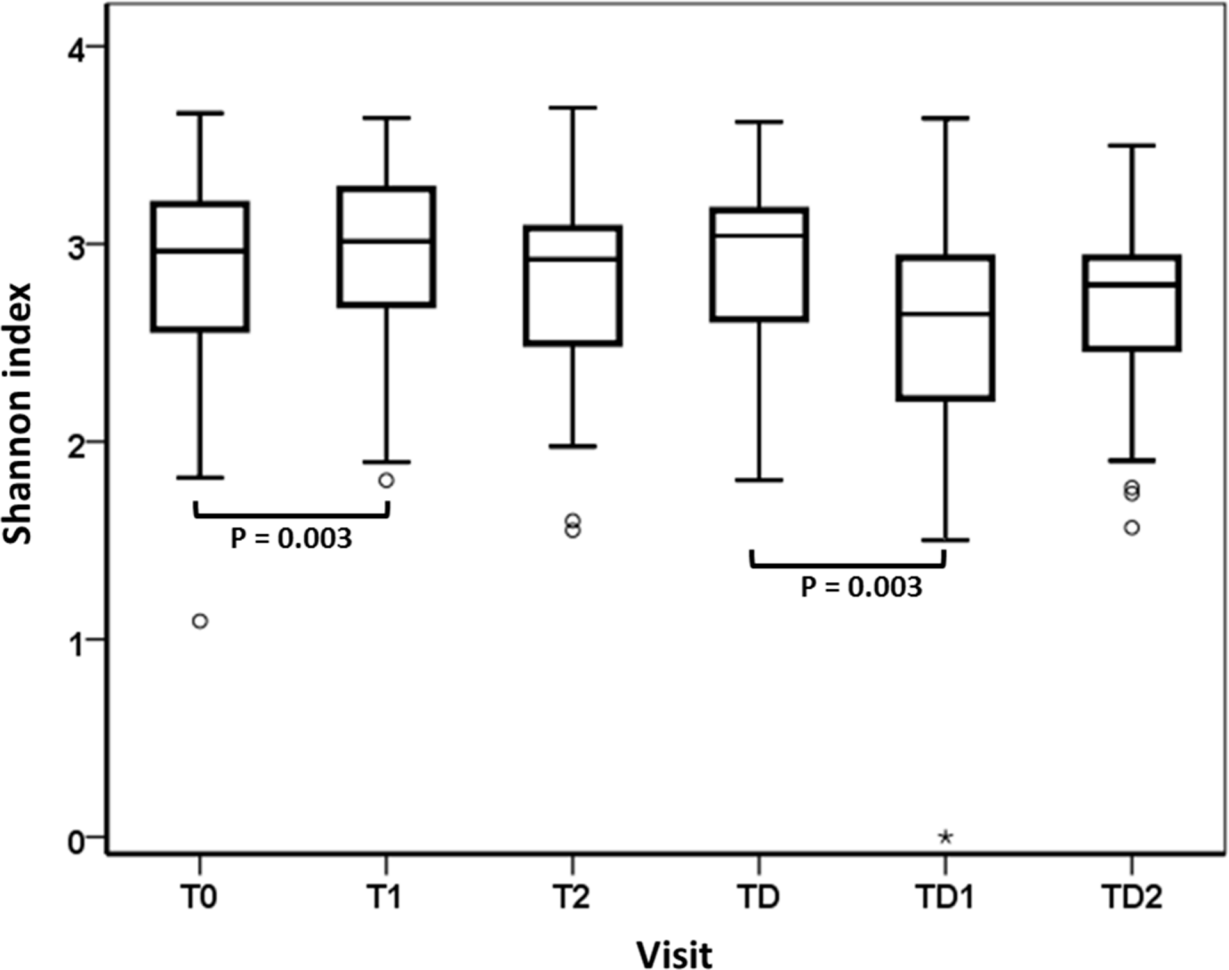
Shannon diversity index for the entire study population per visit. T0; N = 76, T1; N = 73, T2; N = 68, TD; N = 44, TD1; N = 43, TD2; N = 45. Statistical significance (P < 0.05) was determined using the Wilcoxon Signed Ranks test.

Next, we assessed the changes in microbiome of the study population in time. A non-metric multidimensional scaling plot on OTU level was constructed of the individuals (N = 19) whose samples were available from all six time-points. However, no discernable effects of time on the microbiome profiles were found (Fig 4). The microbiome diversity became higher between visit T0 and T1 (P = 0.003) and became lower between visits TD and TD1 (P = 0.003) (Fig 5).

The abundance of the phylum Actinobacteria decreased between visit T0 and T1 (P = 0.043), while the same phylum increased at visits TD1 and TD2 compared to the baseline (P = 0.002, P = 0.006, respectively) (S6A Fig). The phylum Firmicutes had increased in abundance at visits T1 (P = 0.005), TD (P = 0.021) and TD2 (P = 0.035) over visit T0 (S6B Fig). Compared to visit T0, the abundance of Bacteroidetes had decreased in both post-debonding visits: TD1 (P = 0.015) and TD2 (P = 0.025) (S6C Fig). Between visits T0 and TD1, the abundance of Candidate division TM7 decreased (P = 0.031) (S6D Fig), while Fusobacteria decreased from T0 to T2 (P = 0.001) and TD1 (P = 0.001) (S6E Fig). The abundance of Proteobacteria was significantly lower at visit TD compared to the baseline (P = 0.001) (S6F Fig).

**Fig 6:**
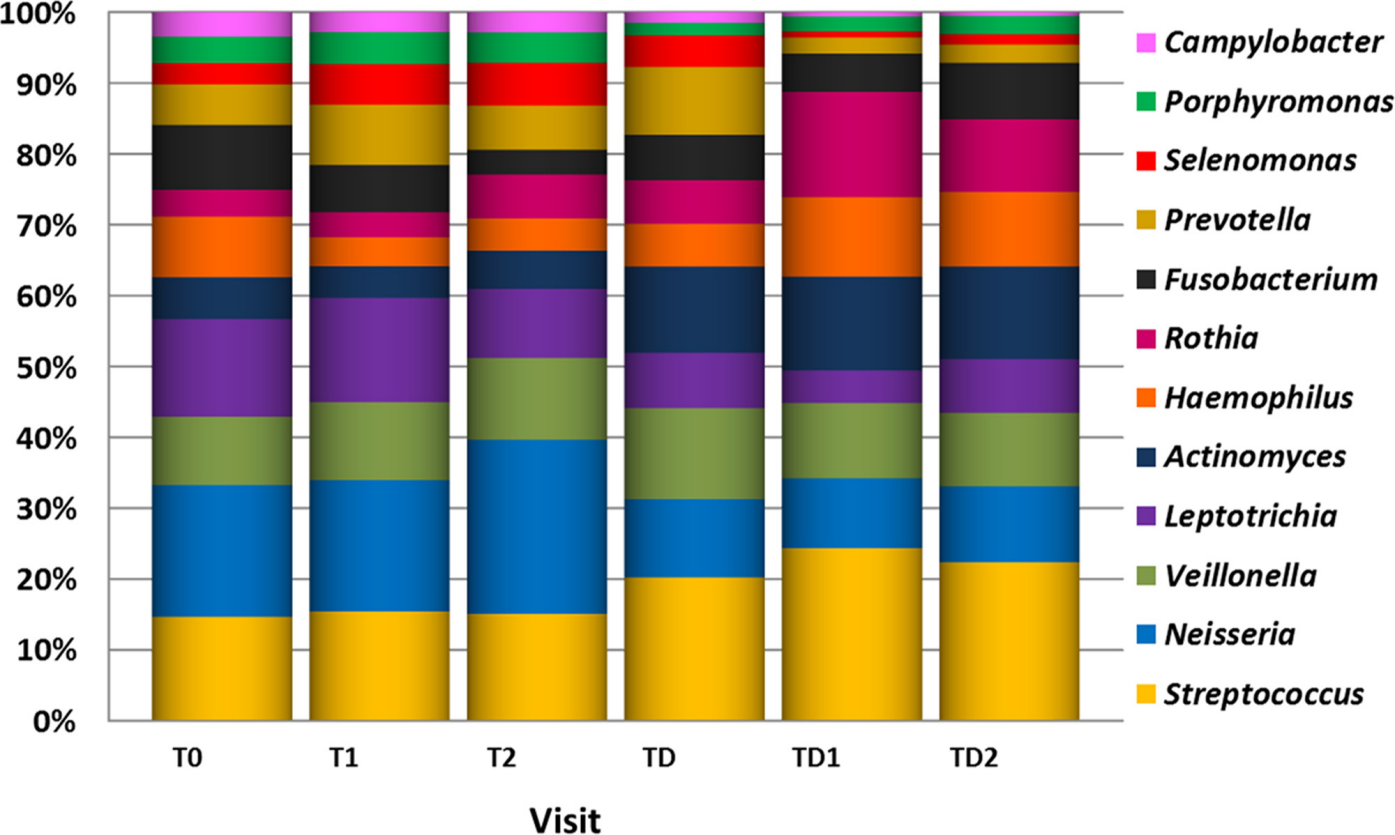
Average proportions of the genera that differed significantly in abundance between one or more of the visits.

Several genera showed significant differences in abundance between the visits (Fig 6). *Streptococcus* became significantly more abundant at visits T1 (P = 0.036), TD (P = 0.025), TD1 (P < 0.001) and TD2 (P = 0.001) compared to the baseline. An increase in abundance from visit TD to TD1 (P = 0.048) was observed as well (S7A Fig). The abundance of *Neisseria* became higher at visit T2 compared to T0 (P = 0.008), while at visits TD and TD1 the abundance became lower compared to visit T0 (P = 0.006, and P = 0.029, respectively). Moreover, the abundance of *Neisseria* increased significantly at visit T2 compared to visit T1 (P = 0.011), yet it was significantly lower again at visit TD (P = 0.018) (S7B Fig). *Actinomyces* had increased significantly at the last three visits when compared to visit T0 (TD: P = 0.004, TD1: P < 0.001 and TD2: P < 0.001) (S7C Fig). Both *Veillonella* (S7D Fig) and *Porphyromonas* (S7E Fig) were only at visit TD significantly more abundant when compared to visit T0 (P = 0.0033 and P = 0.0011, respectively). Additionally, the abundance of *Porphyromonas* decreased significantly between T2 and TD (P = 0.017). For *Leptotrichia*, the abundance became significantly lower at TD1 (P < 0.001) and TD2 (P = 0.037) compared to the baseline (S7F Fig). The abundance of *Campylobacter* had decreased at the last three visits compared to visit T0 (TD: P = 0.033, TD1: P < 0.001 and TD2: P < 0.001) (S7G Fig). At both visits T1 and TD, *Prevotella* had increased in abundance compared to visit T0 (P = 0.004 and P = 0.001, respectively), while at TD1 the abundance had become significantly smaller again (P = 0.010) (S7H Fig). For the genus *Haemophilus*, the only significant increase in abundance was between visits TD and TD1 (P = 0.033) (S7I Fig). The abundance of the genus *Fusobacterium* was significantly lower at T2 and TD1 compared to the baseline (P > 0.001 and P = 0.043, respectively) (S7K Fig). The abundance of *Rothia* was higher in the last three visits compared to the baseline (TD: P = 0.009, TD1: P < 0.001, TD2: P > 0.001) (S7L Fig).

At the OTU level, the abundance of OTU28 (*Actinomyces*) was higher at TD1 (P < 0.001) and TD2 (P = 0.001) compared to visit T0 (S8A Fig). When compared to visit T0, the abundance of OTU65 (*Rothia*) was higher in the last three visits (TD: P 0.009, TD1: P < 0.001, and TD2: P < 0.001) (S8B Fig). In addition, both OTU28 and OTU65 were elevated significantly between visits TD and TD1 (P = 0.049 and P = 0.002, respectively). The abundance of OTU351 (*Streptococcus*) became higher between visits TD and TD1 (P = 0.033) and was significantly higher compared to visit T0 at visit TD1 (P < 0.001) and visit TD2 (P = 0.002) (S8C Fig). In comparison to the baseline, the abundance of OTU398 (*Fusobacterium*) was lower at visit T2 (P < 0.001) and at visit TD1 (P = 0.043) (S8D Fig). The abundance of OTU143 (*Leptotrichia*) decreased significantly between visits TD and TD1 (P = 0.003). Moreover, at visit TD1, the abundance of OTU143 was significantly smaller compared to visit T0 (P = 0.007) (S8E Fig). The abundance of OTU151 (*Campylobacter*) was lower at visit TD compared to visit T2 (P = 0.032) and at TD1 the abundance was lower compared to visit TD (P = 0.001). At both visits TD1 and TD2, the abundance of OTU151 was significantly lower compared to visit T0 (P < 0.001 and P < 0.001, respectively) (S8F Fig). When compared to visit T0, the abundance of OTU302 (*Selenomonas*) had increased at visits T1 (P = 0.002), T2 (P < 0.001) and TD (0.029), while the abundance had decreased at visit TD1 (P = 0.003) (S8G Fig).

## Discussion

The results of our study indicate that the fluoride mouthwash had little effect on the adolescent oral microbiome composition during fixed orthodontic appliance treatment. More pronounced were the microbial changes observed in relation to gingival health status and orthodontic treatment. Yet, the resilience of these adolescent oral communities was noteworthy in regard to the interference caused by the orthodontic treatment, fluoride mouthwash and the physiological changes of puberty itself. There was no observable shift in the composition of the total community in time (Fig 4). A remaining change in abundance was observed for a few genera (Fig 6) and, interestingly, most genera that did increase in abundance in time were associated with a healthy oral cavity.

In this study, an amine fluoride (AmF) combined with stannous fluoride (SnF_2_) mouthwash was used to reduce the amount of demineralization, since fluoride is a well-established anti-caries agent [23] and caries is an infectious bacterial disease. Compliance is regarded as a drawback in studies aiming to observe the effect of a mouthwash. Nonetheless, van der Kaaij *et al*. [11] observed that the use of an AmF/SnF_2_ mouthwash inhibited formation of white spot lesions during this study. Likewise, Øgaard *et al*. [24] observed that there was no difference in white spot lesions before and after orthodontic treatment of patients using an AmF/SnF_2_ mouthwash. Madléna *et al*. [25] observed a decrease in plaque index, gingival index and bleeding on probing within one month in orthodontic patients using AmF/SnF_2_ toothpaste, regardless if the toothpaste was combined with an AmF/SnF_2_ mouthrinse. Van Loveren *et al*. [26] did observe dental plaque shifting towards less acidogenic plaque, yet there was no significant difference in bacterial composition after the use of AmF/SnF_2_ products compared to fluoride-free periods. This is similar to our findings, as we did not observe a clear effect of the fluoride mouthwash on the microbial composition. Although it is suggested that fluoride has antibacterial properties, its main effect appears to be on the demineralization and remineralization processes in the oral cavity [27–30]

We did observe that the abundance of several bacterial taxa was associated with the gingival health status of the subjects. Gingivitis during orthodontic treatment is presumably related to plaque accumulation caused by the newly created retention sites and consequently impaired oral hygiene [5]. Yet, it is not only the orthodontic treatment that is related to the onset of gingivitis in these subjects, for ‘puberty itself’ is also associated with increased gingivitis [31–33]. Generally, orthodontic treatment takes place during adolescence, as was the case in our study. During this period, the human body experiences many (e.g. behavioral and hormonal) changes [34].

The exact reason why gingivitis becomes prevalent in this age-group is unclear but hormonal changes are likely to play a part. Our study did not include a control group of adolescents that did not receive orthodontic treatment. Therefore it is difficult to discern which microbial changes are related to the orthodontic treatment, and which ones to the onset of puberty. Thus far, most studies regarding the (changes in the) oral microbiome during adolescence or orthodontic treatment have focused on a limited number of bacteria, due to the nature of their techniques.

The use of an open ended molecular approach allowed us to detect Candidate division TM7 (and OTUs 55, 171, 355) (S3B and S5D–S5F Figs). Next-generation sequencing has demonstrated that these bacteria, of which only recently a member was grown as a pure laboratory culture [35], are widespread in the human oral cavity [36]. Crielaard *et al*. [37] reported that Candidate division TM7 increased with advancing age, in a study regarding children aged 3–18 years. Duran-Pinedo *et al*. [38] presumed a role for Candidate division TM7 in periodontitis. We found Candidate division TM7 to be associated with gingivitis, in accordance with Huang *et al*. [39].

Interestingly, we observed the presence of the genus *Derxia* (S4D Fig), although low in abundance in our study population, to be related to a healthy state of the gingiva. Members of this genus are known to fix nitrogen in different environmental habitats [40, 41]. Recently *Derxia* has been observed as a member of the human (and canine) oral cavity [42–44], yet its role in this particular environment remains to be elucidated.

Well-known inhabitants of the oral cavity are members of the genus *Prevotella*; often associated with an unhealthy state of the periodontium [45]. Moreover, an increase of *Prevotella intermedia* has been associated with orthodontic treatment [9, 14, 46]. In addition, van Gastel *et al*. [46] observed a decrease of *P*. *intermedia* after the removal of the orthodontic appliances. This coincides with our finding of the abundance of the genus *Prevotella* (S7H Fig). Hence, there appears to be an association between orthodontic treatment and the prevalence of *Prevotella*, although Choi *et al*. [7] did not find a significant decrease of *Prevotella* after orthodontic treatment was ended. This discrepancy might be due to difference in sampling sites or detection techniques.

In this study, we found that the genus *Actinomyces* increased with time (S7C Fig), while OTU28 (*Actinomyces naeslundii*) increased mainly after debonding (S8A Fig). According to Delaney *et al*. [47] the levels of *Actinomyces naeslundii* are higher in prepubertal subjects compared to postpubertal subjects. Gusberti *et al*. [48] observed that the levels of the species *Actinomyces odontolyticus* elevate during puberty. Tanner *et al*. [49] found *Actinomyces* sp. to be associated with gingivitis, whereas Tsuruda *et al*. [50] observed a relation between *Actinomyces* species and healthy pubertal children. These diverse findings indicate that the role of *Actinomyces* in the oral microbiome cannot be determined on genus level, yet it does not explain the contradictory findings of the study by Delaney *et al*. [47] and our own results. Although sampling site and used technique might again be of influence.

The genus *Veillonella* had previously been shown to increase during adolescence [37, 51]. In this study population however, the abundance of *Veillonella* remained stable throughout time (S7D Fig). In addition, the abundance of *Veillonella* was not significantly different between the two mouthwash groups or between the healthy and gingivitis groups.

Both the genus *Campylobacter* (S7G Fig) and OTU151 (*Campylobacter gracilis*) (S8F Fig) decreased with time. A similar pattern of decrease has been observed for *Campylobacter rectus* [7–9]. This decrease could be explained primarily by the reduction of retention sites due to the alignment of the teeth and secondly by the removal of the orthodontic fixed appliances, causing an additional loss of retention sites.

A similar decrease in time was observed for the genera *Porphyromonas* (S7E Fig) and *Selenomonas* (S7J Fig). Additionally, we found that *Porphyromonas* (S4B Fig), *Selenomonas* (S4A Fig) and OTU302 (*Selenomonas*) (S5G Fig) were associated with gingivitis. Members of both these genera are among the main periodontal pathogens [39, 52]. Therefore their decrease in time might be considered desirable. Why they decrease in time, if it is e.g. the reduction in retention sites through alignment of the teeth or hormonal changes in the host, remains unclear.

*Neisseria* became lower in abundance during the advancement of the visits (S7B Fig), in agreement with Moore *et al*. [51], who found this genus to be more associated with prepubertal children than older children. Thus far, most studies investigating the oral microbiome during orthodontic treatment or puberty did not target members of the genus *Neisseria*. Nonetheless, Tanner *et al*. [49] found *Neisseria elongata* to be associated with reduced gingivitis in orthodontic patients. They made the same observation for *Fusobacterium periodonticum*.

Tsuruda *et al*. [50] found *Fusobacterium* sp. to be more abundant in pubertal children with gingivitis compared to healthy children. *Fusobacterium nucleatum* is regarded as a bridging organism in the formation of dental biofilms [53]. This might explain our observation that the genus *Fusobacterium* decreases during the orthodontic treatment, yet increases again in time (S7K Fig). The additional retention sites created by the brackets leave *Fusobacterium* superfluous in the formation of biofilms. On the other hand, Wojcicki *et al*. [54] found that *Fusobacterium* sp. was lower in their circumpubertal group compared to a younger and older test group, suggesting that the presence of *Fusobacterium* sp. is influenced by the physiological maturity of the host.

In contrast to *Fusobacterium*, the abundance of the genus *Streptococcus* (S7A Fig) and OTU351 (*Streptococcus*) (S8C Fig) showed an increase in time without decreasing first. Increase in *Streptococcus* abundance in puberty has been observed before [51], although we cannot identify this member of the genus *Streptococcus* on species level, we speculate that it is associated with a healthy state of the gingiva.

*Haemophilus* (S4E Fig), *Rothia* (S4F Fig) and OTU65 (*Rothia*) (S5A Fig) were associated with a healthy state of the gingiva as well. Their increase after debonding appeared to coincide with the decrease in gingivitis after debonding (Fig 2). Members of these two genera were usually not included as target micro-organisms in studies of the oral microbiome during puberty or orthodontic treatment. Although the role of *Haemophilus* in health and disease of the oral cavity remains somewhat ambiguous, *Rothia* is generally associated with health [15, 55].

In conclusion, the effects of the fluoride mouthwash on the adolescent microbiome were indiscernible and promoted neither health nor disease associated bacterial growth. Yet, van der Kaaij *et al*. [11] did observe fewer demineralizations in subjects using the fluoride mouthwash compared to those using the placebo. Thus, the use of a fluoride mouthwash during orthodontic treatment might be beneficial for the health status of the oral cavity.

Nevertheless, we did observe changes in the abundance of various bacteria. In general, the bacteria that were associated with periodontal pathogenesis decreased in abundance in time, while the abundance of the health related bacteria increased, suggesting that orthodontic treatment during puberty does not have a lasting negative effect on the gingival health status. Still, the lack of an age-related control group not receiving orthodontic treatment precludes us from making a clear distinction between microbial changes instigated by puberty and the effects on the oral ecology caused by orthodontic treatment with fixed appliances. A future study including such a control group would be necessary to determine which microbial changes are truly caused by the presence of orthodontic appliances, allowing for the maintenance of a healthy oral microbiome during orthodontic treatment.

## Supporting Information

S1 Fig Difference in abundance of the genus *Fusobacterium* between mouthwash groups per visit. The read count is displayed on the y-axis. Mouthwashes were administered between visits T0 and TD. Statistical significance (P < 0.05) was determined using the Mann-Whitney test between the two groups per visit, or the Wilcoxon Signed Ranks test within the same group between different visits. The boxes represent the median and interquartile range (IQR), the whiskers represent the minimum and maximum values. Outliers more than 1.5x IQR are depicted by ○, and more than 3x IQR by ★.
(TIF)

S2 Fig Difference in abundance of OTU381 (*Kingella*) between mouthwash groups per visit. The read count is displayed on the y-axis. Mouthwashes were administered between visits T0 and TD. Statistical significance (P < 0.05) was determined using the Mann-Whitney test between the two groups per visit, or the Wilcoxon Signed Ranks test within the same group between different visits. The boxes represent the median and IQR, the whiskers represent the minimum and maximum values. Outliers more than 1.5x IQR are depicted by ○, and more than 3x IQR by ★.
(TIF)

S3 Fig Difference in abundance of the phyla Bacteroidetes (A), TM7 (B) and Fusobacterium (C) based on gingival health status per visit. The read count is displayed on the y-axis. Statistical significance (P < 0.05) was determined using the Mann-Whitney test. The boxes represent the median and IQR, the whiskers represent the minimum and maximum values. Outliers more than 1.5x IQR are depicted by ○, and more than 3x IQR by ★.
(TIF)

S4 Fig Difference in abundance of the genera *Selenomonas* (A), *Porphyromonas* (B), *Johnsonella* (C), *Derxia* (D), *Haemophilus* (E) and *Rothia* (F) based on gingival health status per visit. The read count is displayed on the y-axis. Statistical significance (P < 0.05) was determined using the Mann-Whitney test. The boxes represent the median and IQR, the whiskers represent the minimum and maximum values. Outliers more than 1.5x IQR are depicted by ○, and more than 3x IQR by ★.
(TIF)

S5 Fig Difference in OTU abundance based on gingival health status per visit. The read count is displayed on the y-axis. Statistical significance (P < 0.05) was determined using the Mann-Whitney test. The boxes represent the median and IQR, the whiskers represent the minimum and maximum values. Outliers more than 1.5x IQR are depicted by ○, and more than 3x IQR by ★.
(TIF)

S6 Fig Difference in phylum abundance between visits for Actinobacteria (A), Firmicutes (B), Bacteroidetes (C), TM7 (D), Fusobacteria (E) and Proteobacteria (F). The read count is displayed on the y-axis. Statistical significance (P < 0.05) was determined using the Wilcoxon Signed Ranks test. The boxes represent the median and IQR, the whiskers represent the minimum and maximum values. Outliers more than 1.5x IQR are depicted by ○, and more than 3x IQR by ★.
(TIF)

S7 Fig Difference in genus abundance between visits. The read count is displayed on the y-axis. Statistical significance (P < 0.05) was determined using the Wilcoxon Signed Ranks test. The boxes represent the median and IQR, the whiskers represent the minimum and maximum values. Outliers more than 1.5x IQR are depicted by ○, and more than 3x IQR by ★.
(TIF)

S8 Fig Difference in OTU abundance between visits. The read count is displayed on the y-axis. Statistical significance (P < 0.05) was determined using the Wilcoxon Signed Ranks test. The boxes represent the median and IQR, the whiskers represent the minimum and maximum values. Outliers more than 1.5x IQR are depicted by ○, and more than 3x IQR by ★.
(TIF)

S1 Table BLAST results of the OTUs. (PDF)

S2 Table Number of subjects per group per visit. (PDF)
