# Supplementary figures and images for: The Effect of Fixed Orthodontic Appliances and Fluoride Mouthwash on the Oral Microbiome of Adolescents – A Randomized Controlled Clinical Trial
Source: PLoS One. 2015 Sep 2;10(9):e0137318. doi: 10.1371/journal.pone.0137318 (PMC4558009; doi:10.1371/journal.pone.0137318)

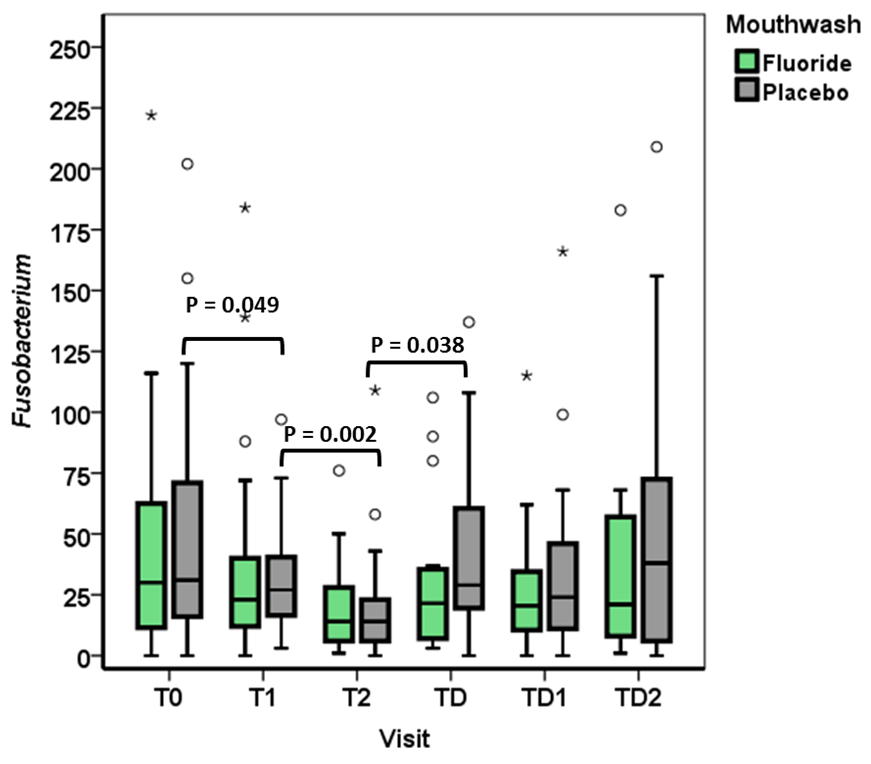

Supplement: S1 Fig — The read count is displayed on the y-axis. Mouthwashes were administered between visits T0 and TD. Statistical significance (P < 0.05) was determined using the Mann-Whitney test between the two groups per visit, or the Wilcoxon Signed Ranks test within the same group between different visits. The boxes represent the median and interquartile range (IQR), the whiskers represent the minimum and maximum values. Outliers more than 1.5x IQR are depicted by ○, and more than 3x IQR by ★. (TIF) [file pone.0137318.s001.tif]

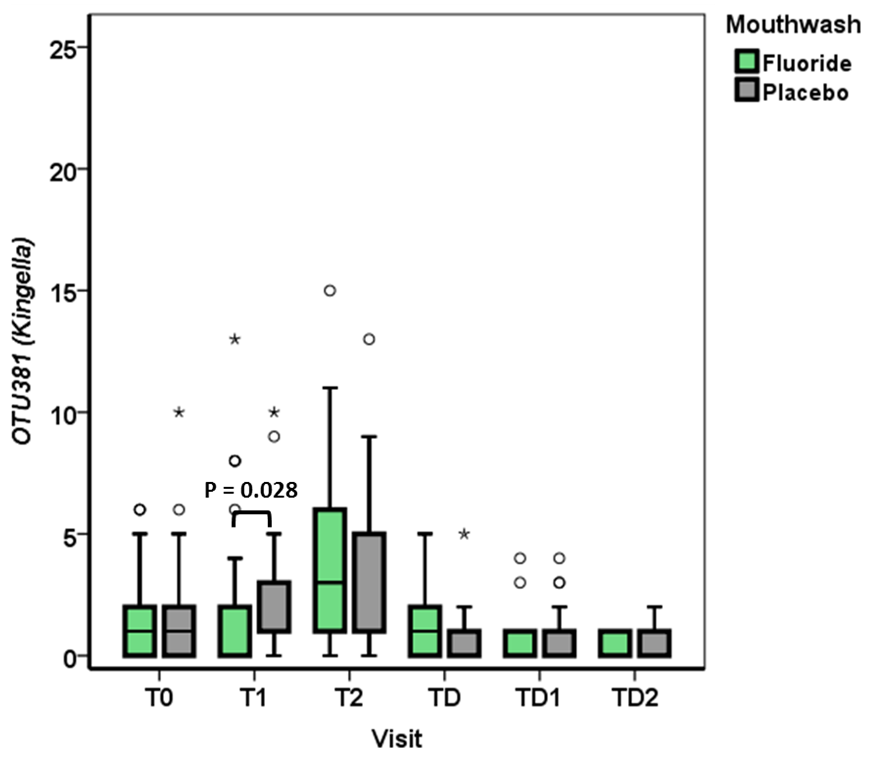

Supplement: S2 Fig — The read count is displayed on the y-axis. Mouthwashes were administered between visits T0 and TD. Statistical significance (P < 0.05) was determined using the Mann-Whitney test between the two groups per visit, or the Wilcoxon Signed Ranks test within the same group between different visits. The boxes represent the median and IQR, the whiskers represent the minimum and maximum values. Outliers more than 1.5x IQR are depicted by ○, and more than 3x IQR by ★. (TIF) [file pone.0137318.s002.tif]

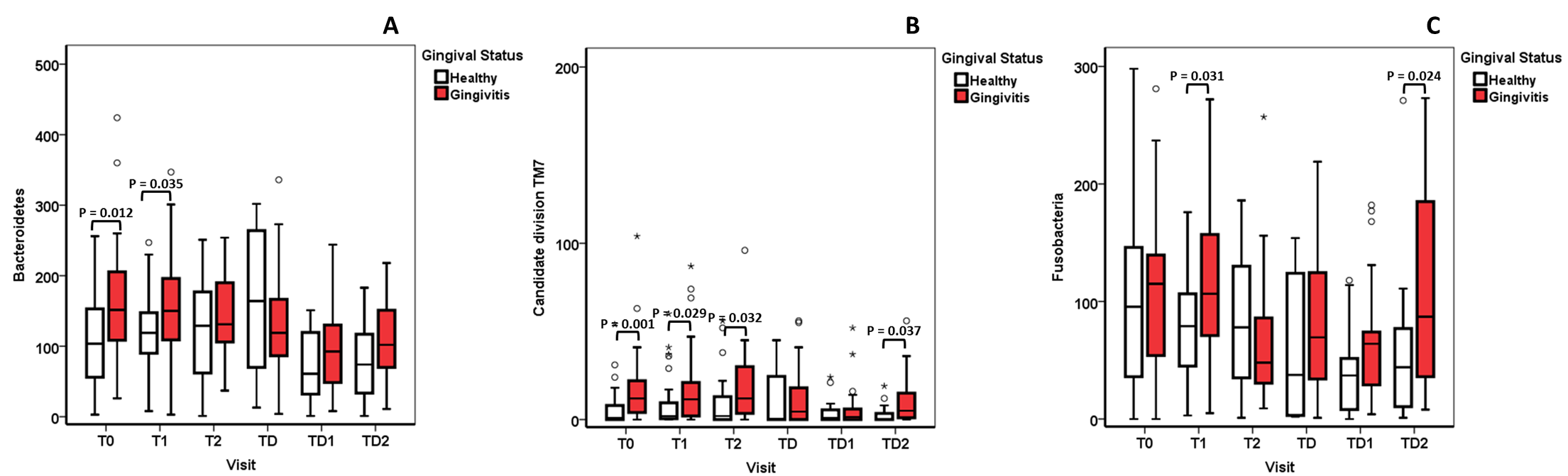

Supplement: S3 Fig — The read count is displayed on the y-axis. Statistical significance (P < 0.05) was determined using the Mann-Whitney test. The boxes represent the median and IQR, the whiskers represent the minimum and maximum values. Outliers more than 1.5x IQR are depicted by ○, and more than 3x IQR by ★. (TIF) [file pone.0137318.s003.tif]

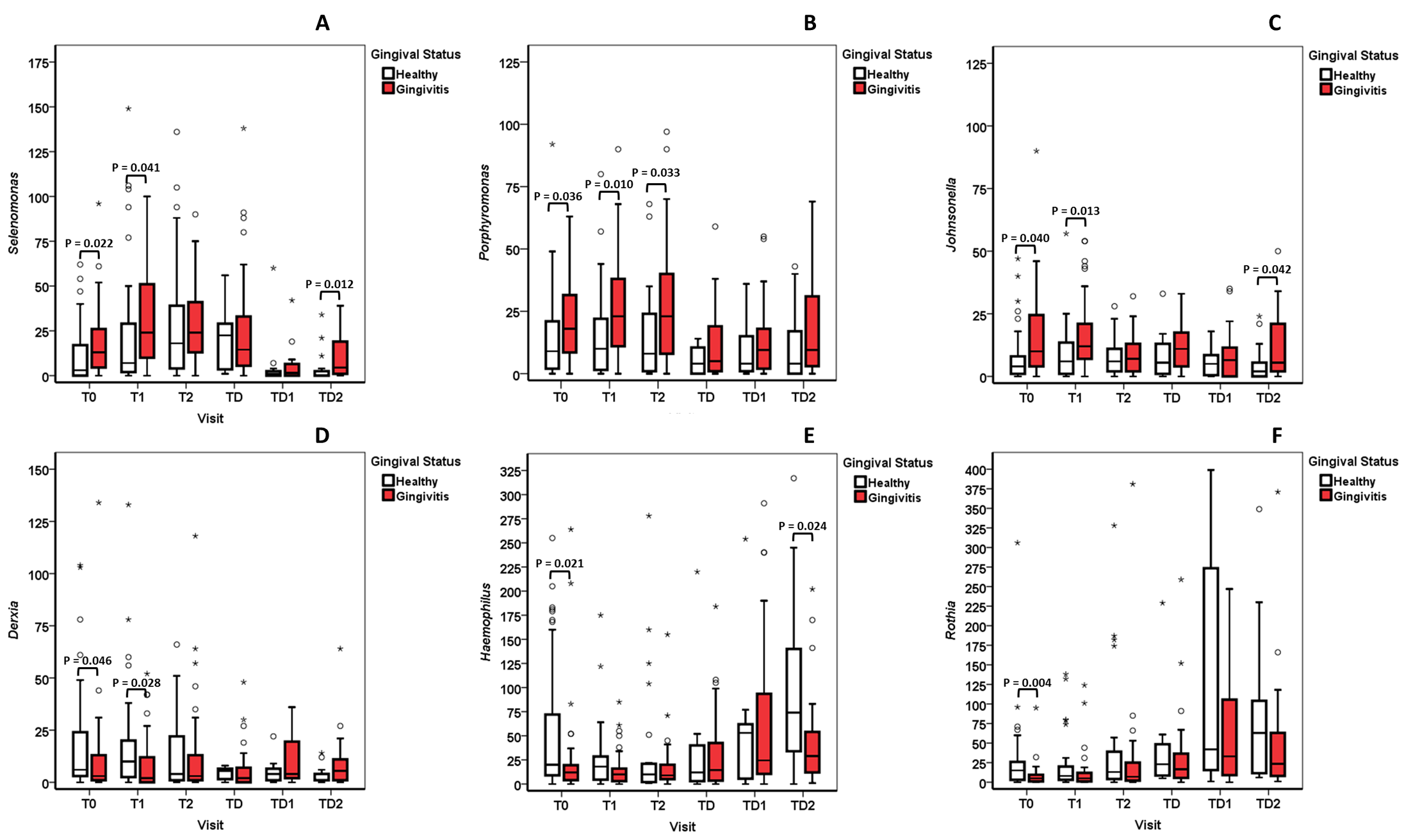

Supplement: S4 Fig — The read count is displayed on the y-axis. Statistical significance (P < 0.05) was determined using the Mann-Whitney test. The boxes represent the median and IQR, the whiskers represent the minimum and maximum values. Outliers more than 1.5x IQR are depicted by ○, and more than 3x IQR by ★. (TIF) [file pone.0137318.s004.tif]

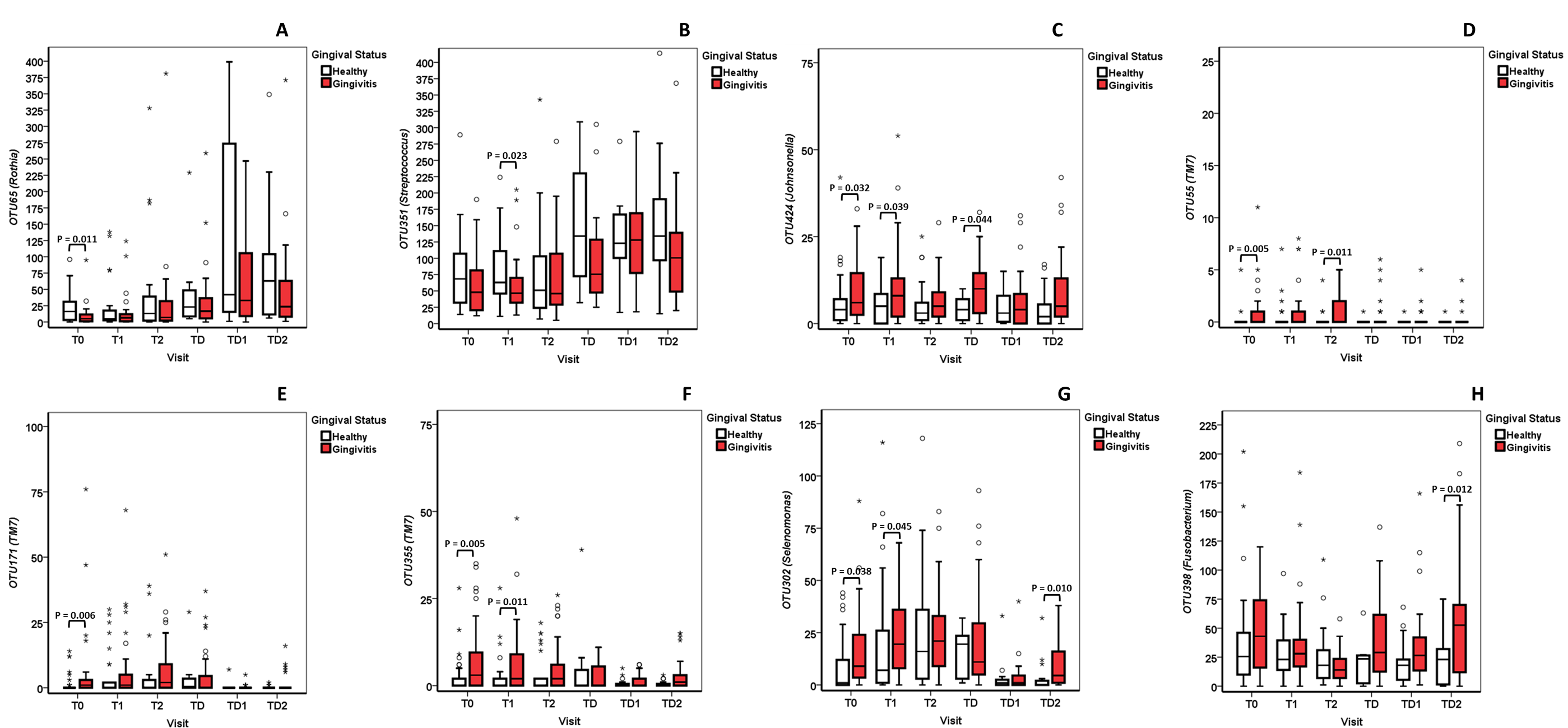

Supplement: S5 Fig — The read count is displayed on the y-axis. Statistical significance (P < 0.05) was determined using the Mann-Whitney test. The boxes represent the median and IQR, the whiskers represent the minimum and maximum values. Outliers more than 1.5x IQR are depicted by ○, and more than 3x IQR by ★. (TIF) [file pone.0137318.s005.tif]

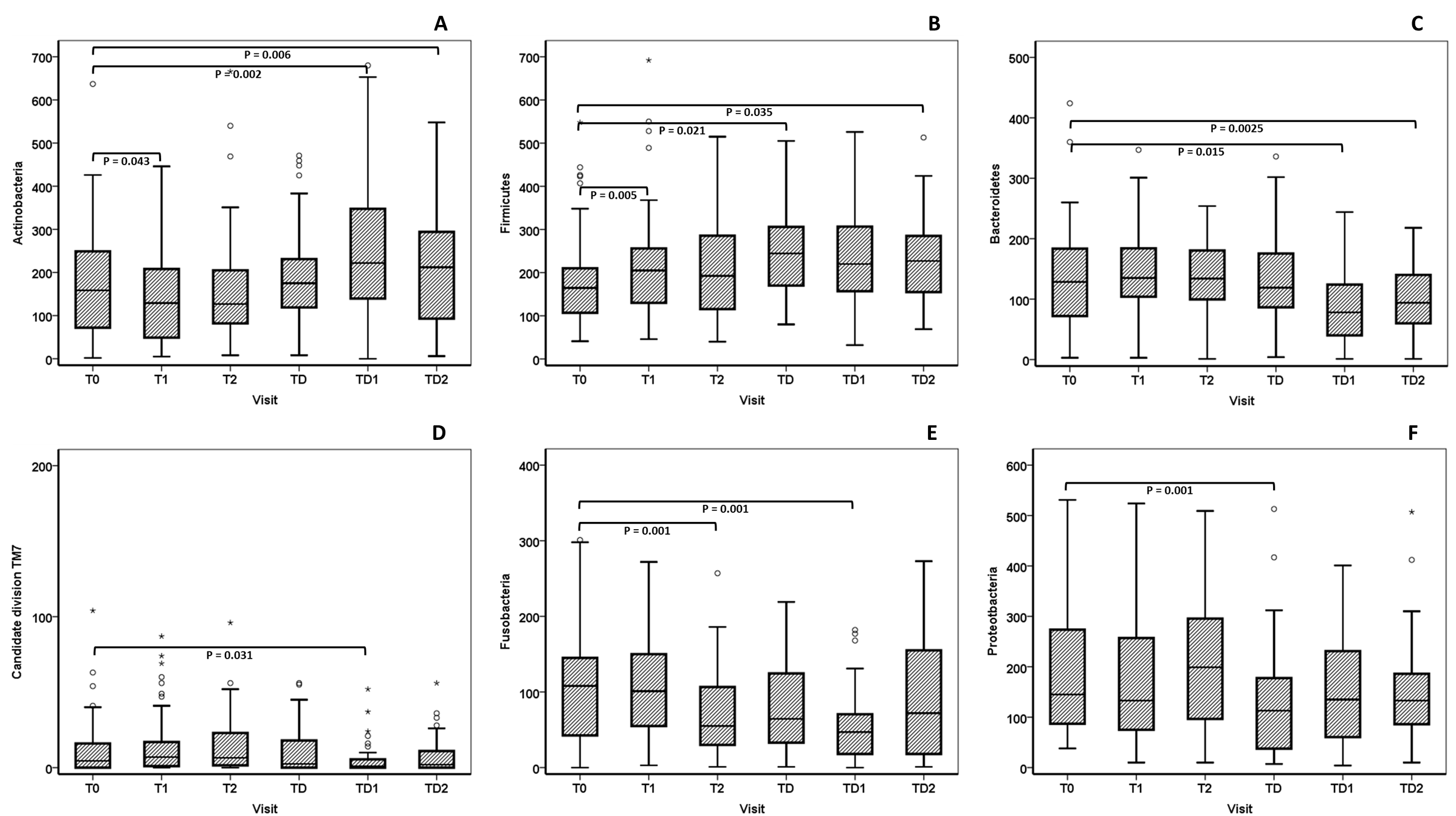

Supplement: S6 Fig — The read count is displayed on the y-axis. Statistical significance (P < 0.05) was determined using the Wilcoxon Signed Ranks test. The boxes represent the median and IQR, the whiskers represent the minimum and maximum values. Outliers more than 1.5x IQR are depicted by ○, and more than 3x IQR by ★. (TIF) [file pone.0137318.s006.tif]

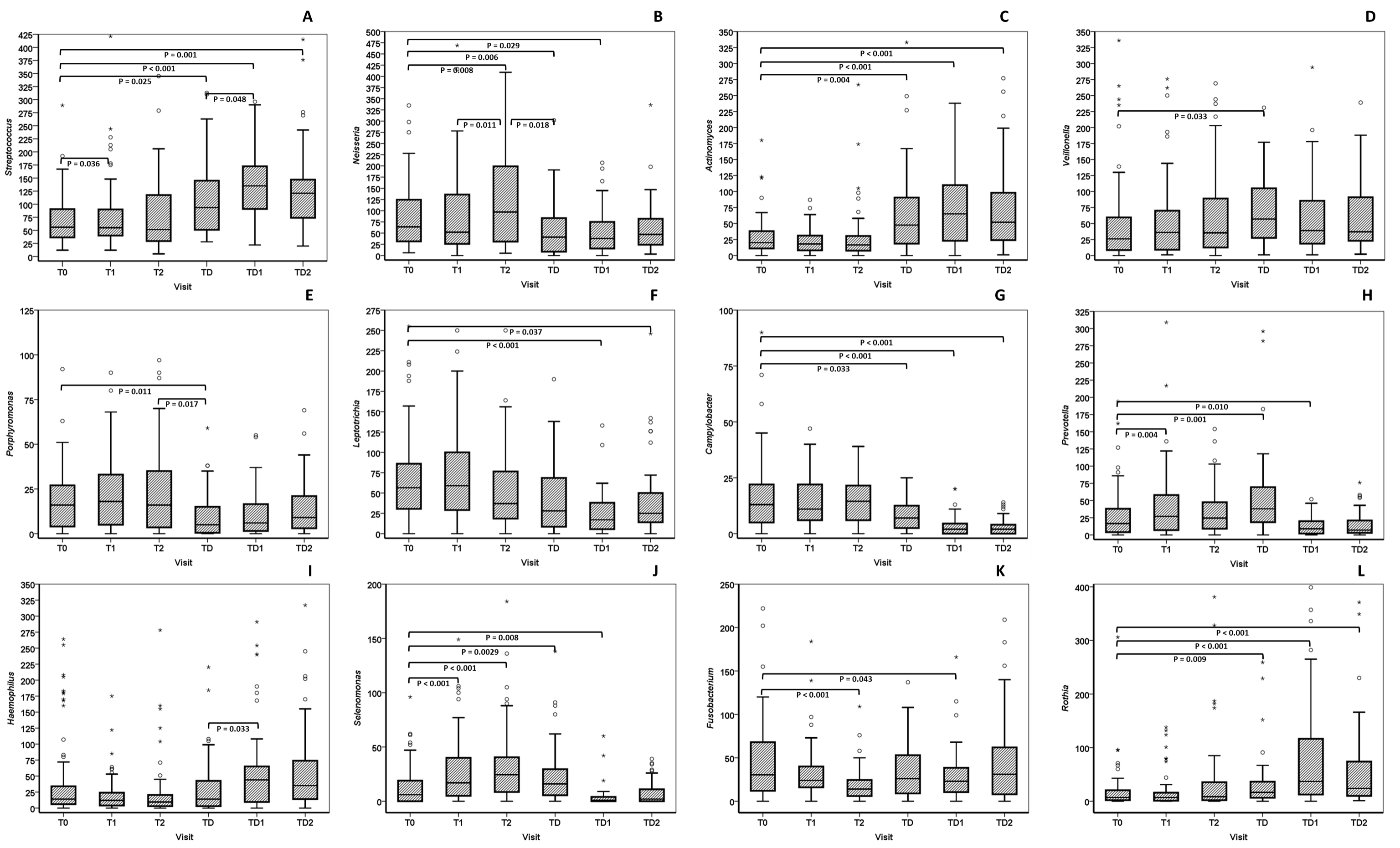

Supplement: S7 Fig — The read count is displayed on the y-axis. Statistical significance (P < 0.05) was determined using the Wilcoxon Signed Ranks test. The boxes represent the median and IQR, the whiskers represent the minimum and maximum values. Outliers more than 1.5x IQR are depicted by ○, and more than 3x IQR by ★. (TIF) [file pone.0137318.s007.tif]

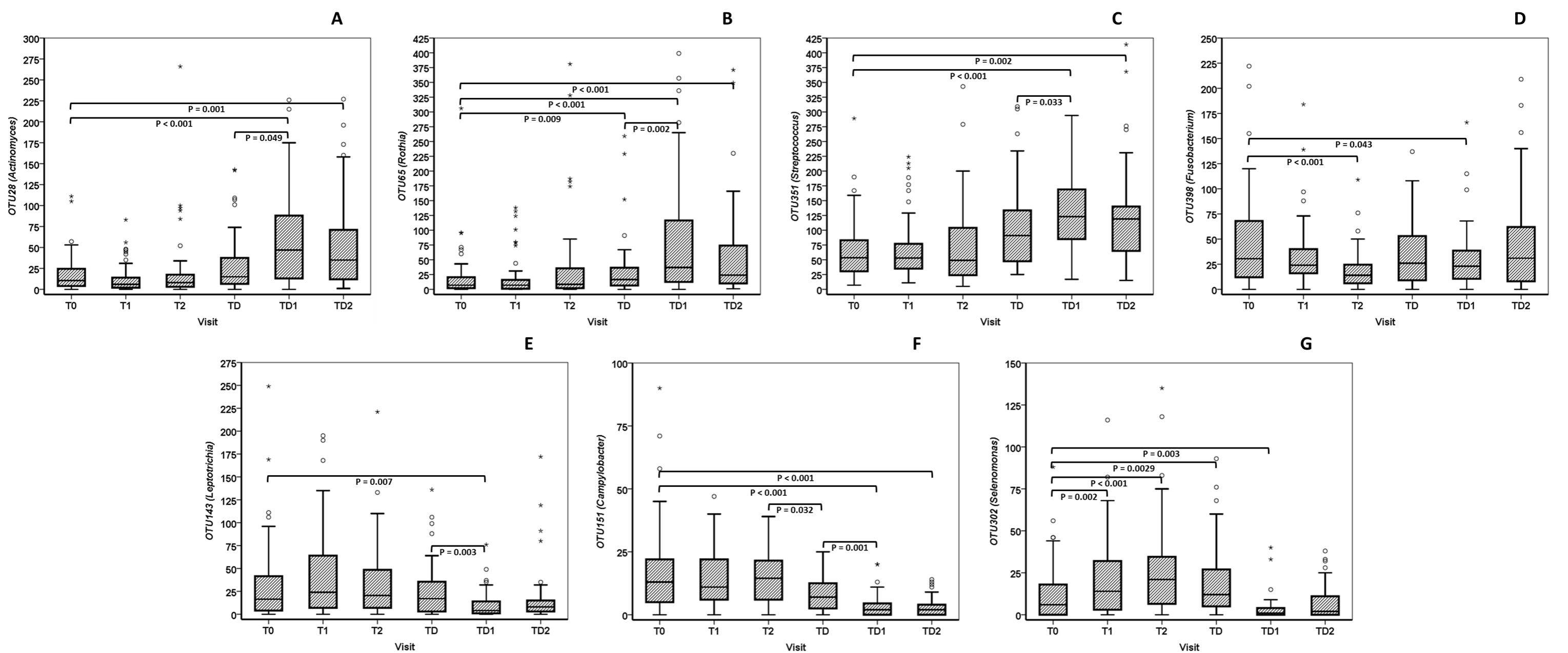

Supplement: S8 Fig — The read count is displayed on the y-axis. Statistical significance (P < 0.05) was determined using the Wilcoxon Signed Ranks test. The boxes represent the median and IQR, the whiskers represent the minimum and maximum values. Outliers more than 1.5x IQR are depicted by ○, and more than 3x IQR by ★. (TIF) [file pone.0137318.s008.tif]
