# Supplementary material for: The Effect of Fixed Orthodontic Appliances and Fluoride Mouthwash on the Oral Microbiome of Adolescents – A Randomized Controlled Clinical Trial
Source: PLoS One. 2015 Sep 2;10(9):e0137318. doi: 10.1371/journal.pone.0137318 (PMC4558009; doi:10.1371/journal.pone.0137318)
Supplement: S1 Table — (PDF) [file pone.0137318.s009.pdf]

S1 Table. BLAST results of the OTUs

#OTU	Description	E-value	Accession
**0**	Catonella morbi clone _Z022 16S ribosomal RNA gene, partial sequence	0.0	GU407024
**2**	Bacteroides cf. forsythus oral clone BU063 16S ribosomal RNA gene, partial sequence	0.0	AY008308
**12**	Porphyromonas catoniae strain JCM 13863 16S ribosomal RNA gene, partial sequence	0.0	NR_113082
**28**	Actinomyces naeslundii 16S rRNA gene, strain CCUG 33914	0.0	AJ234048
**40**	Bacteroidetes bacterium 'Oral Taxon 274' strain F0058 16S ribosomal RNA gene, partial sequence	0.0	FJ577256
**54**	Capnocytophaga ochracea FDC 7b 16S ribosomal RNA gene, partial sequence	9e-178	U41354
**55**	TM7 phylum sp. canine oral taxon 322 clone 1C049 16S ribosomal RNA gene, partial sequence	4e-166	JN713492
**65**	Rothia dentocariosa ATCC 17931, complete genome	0.0	CP002280
**69**	Capnocytophaga sp. AHN9576 16S ribosomal RNA gene, partial sequence	0.0	DQ012327
**114**	Centipeda periodontii strain HB-2 16S ribosomal RNA, partial sequence	0.0	AF458222
**143**	Leptotrichia wadei strain AGU20 16S ribosomal RNA gene, partial sequence	0.0	GU561362
**151**	Campylobacter gracilis strain ATCC 33236 16S ribosomal RNA gene, partial sequence	0.0	NR_118516
**157**	Neisseria sicca strain ATCC 29256 16S ribosomal RNA gene, complete sequence	0.0	NR_121688
**171**	TM7 bacterium human oral taxon HOT-869 clone 4W02 16S ribosomal RNA gene, partial sequence	0.0	KM018321
**185**	Actinomyces dentalis strain R18165 16S ribosomal RNA gene, complete sequence	0.0	NR_025633
**218**	Prevotella saccharolytica strain D080A-01 16S ribosomal RNA gene, partial sequence	0.0	FJ825150
**229**	Capnocytophaga sputigena strain NF10-3338 16S ribosomal RNA gene, partial sequence	0.0	JF422019
**246**	Prevotella sp. canine oral taxon 298 clone ZY032 16S ribosomal RNA gene, partial sequence	6e-150	JN713465
**271**	Cardiobacterium hominis 16S ribosomal RNA gene, partial sequence	0.0	AY360343
**289**	Selenomonas sputigena strain ATCC 35185 16S ribosomal RNA gene, complete sequence	0.0	NR_074905
**302**	Selenomonas noxia strain ATCC 43541 16S ribosomal RNA gene, partial sequence	0.0	NR_028796
**303**	Capnocytophaga leadbetteri strain AHN8730 16S ribosomal RNA gene, partial sequence	0.0	DQ012358
**306**	TM7 phylum sp. canine oral taxon 363 clone 2A026 16S ribosomal RNA gene, partial sequence	9e-128	JN713533
**326**	Leptotrichia sp. PG10 16S ribosomal RNA gene, partial sequence	0.0	GU561363
**332**	Bergeyella sp. AF14 16S ribosomal RNA gene, partial sequence; 16S-23S ribosomal RNA intergenic spacer, complete sequence; and 23S ribosomal RNA gene, partial sequence	0.0	DQ241813
**345**	Lachnospiraceae bacterium canine oral taxon 346 clone 1O089 16S ribosomal RNA gene, partial sequence	4e-151	JN713515
**347**	Prevotella micans strain F0438 16S ribosomal RNA gene, partial sequence	0.0	HM596284
**351**	Streptococcus sp. VT 162, complete genome	0.0	CP007628
**355**	TM7 phylum sp. oral taxon 348 clone BN036 16S ribosomal RNA gene, partial sequence	0.0	GQ422738
**370**	Candidatus Prevotella conceptionensis strain 9403948 16S ribosomal RNA gene, partial sequence	0.0	HM587326
**381**	Kingella denitrificans strain ATCC 33394 16S ribosomal RNA gene, complete sequence	0.0	NR_044658
**390**	Leptotrichia buccalis strain GEJ9 16S ribosomal RNA gene, partial sequence	0.0	GU561361
**398**	Fusobacterium nucleatum strain FDC 364 16S ribosomal RNA gene, partial sequence	0.0	KM023647
**411**	Leptotrichia buccalis DSM 1135, complete genome	9e-168	CP001685
**424**	Human oral bacterium AC32 16S ribosomal RNA gene, partial sequence	0.0	AF201979
**435**	Actinomyces sp. TeJ5 16S ribosomal RNA gene, partial sequence	0.0	GU561315
**453**	Peptostreptococcaceae bacterium OBRC9 16S ribosomal RNA gene, partial sequence	0.0	HQ616354
**454**	Porphyromonas catoniae strain ATCC 51270 16S ribosomal RNA gene, partial sequence	3e-172	NR_026230

The BLAST results were retrieved on 13 November 2014. Sequences were aligned against the Nucleotide collection (nr/nt) using Megablast. Uncultured or environmental samples were excluded from the search results.
