# Supplementary material for: The Effect of Fixed Orthodontic Appliances and Fluoride Mouthwash on the Oral Microbiome of Adolescents – A Randomized Controlled Clinical Trial
Source: PLoS One. 2015 Sep 2;10(9):e0137318. doi: 10.1371/journal.pone.0137318 (PMC4558009; doi:10.1371/journal.pone.0137318)
Supplement: S2 Table — (PDF) [file pone.0137318.s010.pdf]

S2 Table. Number of subjects per group per visit

Visit	Total n	Mouthwash		Gender	
		Fluoride	Placebo	Male	Female
**T0**	76	34	42	36	40
**T1**	73	34	39	38	35
**T2**	68	27	41	32	36
**TD**	44	16	28	20	24
**TD1**	43	20	23	20	23
**TD2**	45	18	27	22	23
